# Electronic health record–integrated approach for collection of patient-reported outcome measures: a retrospective evaluation

**DOI:** 10.1186/s12913-021-06626-7

**Published:** 2021-06-30

**Authors:** Maggie E. Horn, Emily K. Reinke, Richard C. Mather, Jonathan D. O’Donnell, Steven Z. George

**Affiliations:** 1 Division of Physical Therapy, Department of Orthopaedic Surgery, Duke University, 311 Trent Drive, Durham, NC 27710 USA; 2 Division of Sports Medicine, Department of Orthopaedic Surgery, Duke University, 3475 Erwin Rd, Durham, NC 27705 USA; 3 Duke-Margolis Center for Health Policy, Duke University School of Medicine, Durham, NC USA; 4 Department of Orthopaedic Surgery and Duke Clinical Research Institute, Duke University, 200 Morris Street, Durham, NC 27001 USA

**Keywords:** Electronic health record, Orthopedics, Patient-reported outcome measures, Value-based care

## Abstract

**Background:**

The integration of Patient Reported Outcome Measures (PROMs) into clinical care presents many challenges for health systems. PROMs provide quantitative data regarding patient-reported health status. However, the most effective model for collecting PROMs has not been established. Therefore the purpose of this study is to report the development and preliminary evaluation of the standardized collection of PROMs within a department of orthopedic surgery at a large academic health center.

**Methods:**

We utilized the *Users’ Guide to Integrating Patient*-*Reported Outcomes in Electronic Health Records* by Gensheimer et al., 2018 as a framework to describe the development of PROMs collection initiative. We framed our initiative by operationalizing the three aspects of PROM collection development: Planning, Selection, and Engagement. Next, we performed a preliminary evaluation of our initiative by assessing the response rate of patients completing PROMs (no. of PROMs completed/no. of PROMs administered) across the entire department (18 clinics), ambulatory clinics only (14 clinics), and hospital-based clinics only (4 clinics). Lastly, we reported on the mean response rates for the top 5 and bottom 5 orthopaedic providers to describe the variability across providers.

**Results:**

We described the development of a fully-integrated, population health based implementation strategy leveraging the existing resources of our local EHR to maximize clinical utility of PROMs and routine collection. We collected a large volume of PROMs over a 13 month period (*n* = 10,951) across 18 clinical sites, 7 clinical specialties and over 100 providers. The response rates varied across the department, ranging from 29 to 42%, depending on active status for the portal to the electronic health record (MyChart). The highest single provider mean response rate was 52%, and the lowest provider rate was 13%. Rates were similar between hospital-based (26%) and ambulatory clinics (29%).

**Conclusions:**

We found that our standardized PROMs collection initiative, informed by Gensheimer et al., achieved scope and scale, but faced challenges in achieving a high response rate commensurate with existing literature. However, most studies reported a targeted recruitment strategy within a narrow clinical population. Further research is needed to elucidate the trade-off between scalability and response rates in PROM collection initiatives.

## Background

Patient-reported outcome measures (PROMs) quantify outcomes that are important to patients such as disease symptoms, health-related quality of life, and patient satisfaction [1]. PROMs can be used to support patient-provider decision-making by standardizing how patient’s health status is reported [2]. Moreover, PROMs play an important role in advancing patient-centered care, such as providing information to guide diagnostic and treatment decisions; in value-based health care [3]; and in patient-centered comparative effectiveness trials [4, 5]. Therefore it is vital for health systems to integrate PROMs collection into routine clinical care to improve patient care, engage in shared decision-making, and complete meaningful patient-centered research [6].

Patient-related outcome information is a prerequisite for patient centered care, which has increasingly been recognized as an ethical imperative in modern health care [7]. However, the integration of PROMs into clinical care presents many challenges for health systems [8], and widespread implementation of routine collection has been limited due to clinician, staff, and patient reluctance, inadequate resources to deal with positive or unexpected results, concerns about how the data will be used, and workflow and technology challenges [9]. However, effective patient-centered care requires the routine integration of PROMs [10], and integration of PROMs into the electronic health record (EHR) is fundamental to advancing clinical care by providing quantitative data regarding patient reported patient health status [11].

Before implementation of PROMs in clinical practice, feasibility of patient burden, logistics of workflow impact, display of results, and administration frequency should be carefully evaluated, just as psychometric properties and applicability of the outcomes [12]. Through a collaboration between the International Society for Quality of Life Research and the Patient-Centered Outcomes Research Institute, researchers from Johns Hopkins University developed the *Users’ Guide to Integrating Patient*-*Reported Outcomes in Electronic Health Records* [13]. The guide provides a practical framework and examples for administrators, clinicians, researchers, information technology (IT) professionals, and others wishing to integrate patient-reported outcomes into their EHRs. It offers guidance on 11 key questions related to integration and for each question describes advantages and disadvantages of different options and identifies research gaps. As a follow-up to the *Users’ Guide*, Gensheimer et al. presented the 11 key questions in 3 thematic groups—Planning, Selection, and Engagement—and described the key questions and their important considerations and takeaways for implementation [14]. Addressing and understanding these key issues around PROMs-EHR integration will improve the adoption and implementation PROMs collection in a given health system.

The purpose of this study is to describe the utilization of the thematic framework by Gensheimer et al. [12], informed by the *Users’ Guide* [13], to develop and implement a large-scale, EHR-integrated approach to PROMs collection within the orthopedic surgery department at a large academic health center. We utilized this framework to report our PROMs implementation initiative and framed our approach using the three thematic groups: Planning, Selection, and Engagement. We performed a 13-month retrospective evaluation of the PROMs collection implementation initiative by analyzing the scope, scale, and patient collection response rates associated with this initiative. By doing this, we highlight the barriers and facilitators to implementing best practices for EHR-integrated approach to PROMs collection demonstrating the value proposition for PROM collection initiatives for health systems. Moreover, we were able to evaluate the challenges and opportunities of implementing a large-scale, real-world PROMs collection across a heterogeneous clinical department.

## Methods

### Study setting

The EHR-integrated PROMs collection initiative was conducted in the Department of Orthopaedic Surgery at a university health system in North Carolina. The Department of Orthopaedic Surgery PROMs collection initiative took place across 18 adult clinics (14 ambulatory and 4 hospital-based clinics) and covered 7 specialties (Joint Reconstruction, Spine, Sports Medicine, Trauma, Oncology, Foot and Ankle, and Hand) with over 100 orthopedic providers including surgeons, physician assistants, and nurse practitioners.

In 2017, as part of the Department of Orthopaedic Surgery’s strategic plan, the integration of PROMs collection in routine clinical care was identified as a departmental priority. The previous state of PROMs collection within the department was highly variable and lacked a standardized approach and associated EHR infrastructure. Moreover, access to PROMs data inside and outside the EHR was limited, which hindered a scalable implementation of PROMs collection. Therefore, a multidisciplinary team was assembled to develop a strategy for implementation of standardized PROMs collection within the department. The multidisciplinary team consisted of faculty in the department including clinical researchers with expertise in clinical informatics, data analytics, outcomes, and musculoskeletal research, as well as an orthopedic surgeon and orthopedic practice administrator. Key considerations for the multidisciplinary team when developing the PROMs collection strategy as identified in the strategic plan were cost, scalability, actionability of data, impact on operations, and patient engagement.

### Integration of PROMs in the EHR: planning, selection, and engagement

To assess our strategy for standardized PROMs collection, we discussed the 11 key questions detailed in the *Users’ Guide* [11], as well as Gensheimer et al.’s [14] three thematic groups: Planning, Selection, and Engagement. We utilized this thematic framework to facilitate our discussions regarding our PROMs implementation initiative.

The planning stage for the PROMs implementation involved answering the following key questions: What strategy will be used for PROMs integration? How will the data be governed? Are there ethical and legal issues? How can data from EHR sources be pooled inside and across organizations? The key questions to be addressed during the selection stage for PROMs implementation included: Which populations and patients are most suitable for collection and use of PROMs data, and how can EHRs support identification of suitable patients? Which outcomes are important to measure for a given population? How should candidate PROMs be evaluated? To aid in PROM selection, we administered an electronic survey to identify the PROMs currently being collected and the PROMs they would like to use in the future. Lastly, the providers were asked for what purposes, if any, they would like to use PROMs outside of clinical care. Lastly, the engagement stage was intended for determining how, where, and with what frequency patients would respond to PROMs; how to display PROMs data within the EHR; how clinical teams would act upon PROMs data; and how to train, support, and incentivize clinical teams and patients.

### Evaluation of implementation of PROMs collection initiative

After we completed the planning, selection, and engagement phases of the EHR-integrated PROMs collection initiative, we implemented standardized PROMs collection on November 17, 2017. Approximately 13 months after implementation, we performed a preliminary evaluation of the PROMs collection initiative. The primary metric we were interested in evaluating was the response rate of patients completing PROMs, a key indicator of success for PROMS collection. We reported the total number of PROMs completed and mean response rates (no. of PROMs completed/no. of PROMs administered) at baseline across the entire department (18 clinics), ambulatory clinics only (14 clinics), and hospital-based clinics only (4 clinics). Lastly, we reported on the mean response rates for the top 5 and bottom 5 orthopaedic providers to describe the variability in providers. Our PROMs administration strategy relied on the use of the EHR patient portal, MyChart, for PROMs administration. Patients who did not have access to MyChart prior to their appointment could complete the questionnaires via Welcome Tablets. Welcome tablets are EHR integrated tablets or iPads used to collect PROMs and directly communicate with the patient record. During the implementation period, Welcome tablets were only available for use in 2 clinics and not consistently used to collect PROMs. Therefore, we reported response rates in 1) MyChart active patients who filled out PROMs via patient portal only and 2) all patients (i.e., both MyChart active and not active patients). These metrics were provided to indicate the different denominators corresponding with each collection strategy for calculating response rates. All analyses were performed using STATA SE/15.1 and Tableau.

## Results

### Framework for PROMs collection

#### Planning

Our process and key questions answered during the EHR-integrated PROMs collection initiative are summarized in Table 1. The multidisciplinary group considered several key issues when exploring options for the PROMs collection strategy, including cost, scalability, access to data, and impact on clinical operations and patient burden. Two viable options for PROMs collection were then considered: 1) third-party PROMs collection with EHR integration and 2) full integration of PROMs collection within the existing EHR. The identified advantages to the third-party approach were the access to data for clinicians and researchers outside the EHR, benchmarking with other orthopedic departments and providers, ability to enroll patients in registries, and wide availability of orthopedic-specific outcome measures. The disadvantages to this approach were high annual cost per user, 2-way integration with EHR systems that would require EHR governance approval and result in potentially lengthy implementation, and concerns about clinician adoption within the workflow. The alternative approach (full integration of PROMs collection) presented minimal or no upfront cost for integration beyond time resources by the local IT team. This approach supported scalability for PROMs administration across the department with low perceived burden to clinicians or staff to access PROMs data during the clinical encounter within the existing EHR workflow. The main disadvantage of the full integration approach was the inflexibility of the EHR for customizing PROMs and data visualizations.

**Table 1:** Planning phase of PROMs collection implementation initiative

Process	Key Questions	Options for Implementation	Identified Implementation Strategy
Integration	What strategy will be used for integrating PROMs in EHRs?	Minimal: Staff or clinicians manually enter PROMs data into the EHR (e.g., paper scanning, clinical note entry); many variations of this exist	Full integration approach to PROMs collection
		Third-party: PROMs can be collected through a specific vendor interface and mapped to the EHR into discrete fields.	
		Full integration: PROMs are collected from the patient and reported directly within the EHR	
Governance	How will the PRO-EHR system be governed?	Centralized: PROMs governed by COORDS stakeholder group	Centralized governance by the multidisciplinary group
		Distributed: PROMs governed at the division level	
Ethical considerations	What is the intended use of PROMs data?	Patient care, research, and/or population surveillance	PROMs collection as part of standard of care for primary use in patient care and population health
	How will patient privacy and burden be managed?	Patient information collected as part of standard of care or request informed consent	Safeguards embedded in EHR to protect against redundant data collection and unauthorized access
PROMs data extraction and storage	How can PROMs data from multiple EHRs be pooled?	How can data be structured and stored to support various efforts? Local datamart: PROMs data are stored locally, and a data model is created to outline the PROMs data to be shared across institutions Centralized data warehouse: Data from each local EHR are stored in one location	Local datamart with a data model developed to routinely extract PROMs directly from the EHR along with other clinically relevant data

*COORDS* Comprehensive Outcomes in Orthopaedics and Rehabilitation Data System, *EHR* electronic health record, *PROMs* patient-reported outcome measures

Next, we considered how the data would be governed, as well as ethical matters. A centralized governance model would facilitate standardized implementation of PROMs collection, with an emphasis on coordination of data across multiple divisions and centralized ethical oversight, whereas a distributed model could hinder the standardization of PROMs collection and lead to fragmented collection and alignment of PROMs collection goals. Lastly, the multidisciplinary group discussed how the data should be stored, accessed, and used outside the clinical encounter.

After these considerations, we chose to implement a full EHR integration approach to PROMs collection. The full integration approach required that PROMs are collected from the patient through MyChart and reported directly within the EHR (MaestroCare) for providers. By doing this, providers had the ability to communicate about PROMs and results with patients through the existing patient portal. Moreover, with this approach, our goal was to incorporate the collection of PROMs as part of the standard of care. The governance of PROMs collection was to be initially overseen by the multidisciplinary group and followed by a steering committee of clinicians, operations and IT staff, and researchers within the department after the initial implementation. Our goal in planning this approach was to facilitate improved access to PROMs data for providers during the clinical encounter within the existing EHR workflow. Lastly, the full integration approach of PROMs collection limits patient burden by providing a safeguard against redundant data collection within the EHR that can occur with third-party or minimal integration approaches. In collaboration with the local analytics team, the multidisciplinary group designed a data model prototype to routinely extract PROMs directly from the EHR along with other clinically relevant data and a dashboard for data visualization.

#### Selection of PROMs

The process of identification of outcomes and key questions addressed in the selection of PROMs are detailed in Table 2. Providers surveyed, both operative and non-operative, were most interested in outcomes such as physical function, disability, pain, quality of life and psychosocial disposition. Providers reported PROMs were most important for improving clinical care and to benchmark their patient outcomes and they would like to use PROMs data for quality improvement and research initiatives.

**Table 2:** Selection phase of PROMs collection implementation initiative

Process	Key Questions	Implementation Considerations	Identified Implementation Strategy
Population	How and to whom will PROMs be assigned?	Tailored Approach: PROMs anchored to surgical event, patient condition and/or by clinical division Population-based Approach: PROMs anchored by a singular, common encounter type across the entire department	Population-based approach, PROMs collection anchored by singular, common encounter type: new patient appointment
Outcome Selection	What outcomes are providers most interested in collecting?	Constructs of physical function, disability, pain, quality of life, and psychosocial disposition	Identify measures that capture these outcomes across a diverse group of patients and diagnoses
PROMs Selection	What are the candidate PROMs to measure outcomes of interest?	Region specific or legacy PROMs: involve the use of PROMs related to specific health conditions or diagnosis Generic PROMs: Assess specific outcomes and constructs of interest to majority of providers, regardless of clinical division or patient population (surgical and non-surgical)	Generic PROM

Therefore, we chose to implement a standardized population health-based system (as opposed to a tailored approach) for collecting PROMs that would be inclusive of the different provider types within the department (i.e., surgical and nonsurgical, rehabilitation) and allow for longitudinal measurement of PROMs at set time intervals. The common system that was selected was PROMIS® (Patient-Reported Outcomes Measurement Information System) [15].PROMIS is validated for use in multiple orthopedic populations and is anchored to a t-score metric allowing for consistent interpretation of findings, and it can be mapped to many region-specific or legacy measures [16, 17]. For similar reasons as selecting a population-based approach for PROMs collection (scalability, benchmarking across divisions, and actionability of data), we chose generic measures to be collected across the department. PROMIS measures have multiple domains available to quantify each of the outcomes of interest identified by providers in the orthopedics department. In the initial phases of implementation, we selected 8 PROMIS domains to collect using short forms. This decision was pragmatic and was based on the availability of PROMIS measures available within Epic’s Foundation system, which is a collection of all the functionality developed by former Epic customers, and provider preference for constructs to measure. The selection of common PROMs and domains across the department allowed providers within and across divisions to compare outcomes across all orthopedic patients, and provided the standardization needed to engage in value-based care programs and large, pragmatic trials. The long-term plan was to transition to computer adaptive testing PROMIS tools after preliminary implementation and when this technology was available within our instance of Epic.

#### Engagement

##### Provider engagement

One aim of our initiative was to engage the department in PROMs collection and use of PROMs in clinical care. To this end, we promoted engagement of providers and patients by leveraging the use of the EHR (Epic MaestroCare) and patient portal (Epic MyChart) to administer PROMs to patients and for providers to view and act upon PROMs data at the point of care. Leveraging the EHR and patient portal allowed providers the ability to communicate with patients about PROMs both during and outside the clinical encounter. Providers and clinical staff were educated on PROMs collection methods and provider EHR interaction with the PROMs data through structured presentations and materials.

**Fig. 1:**
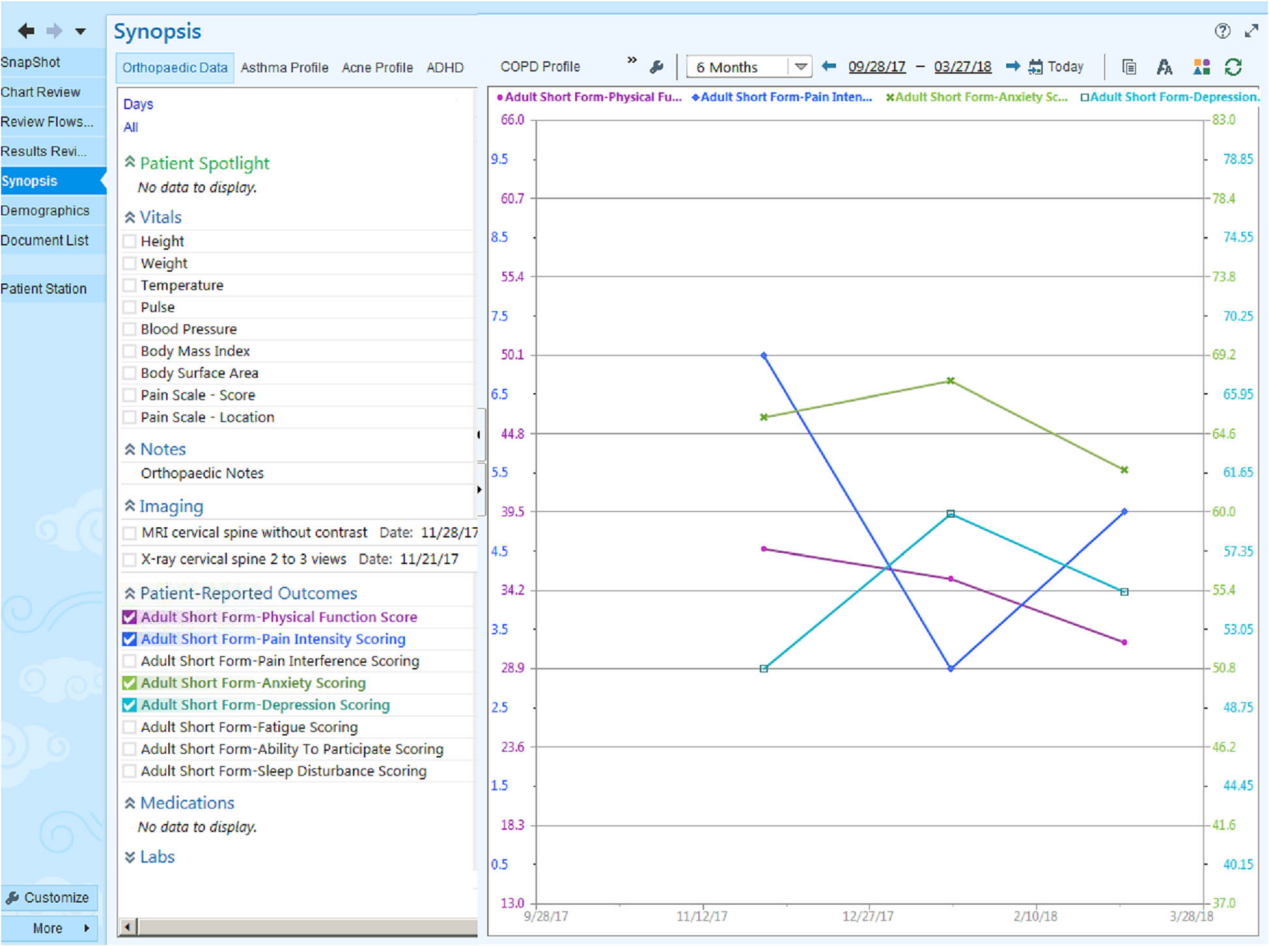
Synopsis activity view of PROMIS data. *Synopsis activity view has been altered to improve visualization of PROM data

To provide feedback on PROMs performance as well as visualization access to patient-level PROMs data, the Epic Synopsis Activity View was created. This allows providers to view trends in PROMs data over time and discuss with patients (Fig. 1). A time-lagged Tableau® dashboard was created to display aggregate PROMs data to be viewed at the provider, clinic location, and division level. Visualization access of standardized PROMs across all divisions allows department members to view and compare data across different patient populations and providers, with a common metric (PROMIS). Providers reported that the synopsis report was helpful for viewing PROMs as well as other clinical data, however for some this was not part of their regular workflow. Providers and PROMIS experts indicated that the strength of the dashboard was the comparison across providers and visualization of PROMs. Providers reported that response rate and regular access to the dashboard was important for continued engagement.

##### Patient engagement

PROMs were administered to patients ahead of their scheduled appointment through MyChart, providing the patients the opportunity to complete the PROMs prior to their appointment. If patients did not complete the PROMs prior to their scheduled appointment, EHR-integrated tablet computers (operating the EPIC Welcome module) were available at limited clinical sites to collect PROMs during the clinical encounter. However, the tablets were not used regularly throughout the implementation phase.

We chose to deploy the PROMs via a questionnaire series, rather than anchored to an appointment type such as all new and return visits. This was the option recommended by our EHR IT team to leverage the reporting capabilities within Epic and to reduce patient fatigue. Moreover, this approach allowed patients to complete the questionnaires outside of their clinical visit, reducing the need and potential burden associated with in-clinic collection but allowing longitudinal measurement. The visit anchor for the questionnaire series was a new patient appointment with an orthopedic provider within the 18 designated MaestroCare departments, which are reflective of the clinical locations. Once a patient completed the first PROM, the anchor rule defined by MaestroCare was met and the PROMIS questionnaire series was initiated. Through MyChart, patients would be messaged reminders to complete the follow-up PROMs. If enabled by the patient, an e-mail reminder would also be sent to patients. Follow-up time points began at 6 weeks after the completion of the initial questionnaire and then quarterly for 1 year. This approach allowed for remote collection of PROMs, aiming to decrease the burden on clinical sites and promote routine patient engagement. See Table 3 for a summary of engagement considerations.

**Table 3:** Engagement phase of PROMs collection implementation initiative

Process	Key Questions	Implementation Considerations	Identified Implementation Strategy
Patient PROMs administration and collection	How and when will PROMs be collected?	Reliance on PROMs collection via clinical encounter: PROMs collection “on every patient, every time” Reliance on PROMs collection via patient portal: Time series approach with initial questionnaire completion as trigger to automation of subsequent questionnaires over time	PROMs collection via patient portal; questionnaire series
Provider visualization of PROMs	How will PROMs be accessed and visualized inside and outside the EHR?	Patient-level visualization: flowsheets, review flowsheets, clinical note and/or Synopsis Activity View Aggregate responses: Epic reporting or Tableau Dashboards	PROMs visualization at the patient level primarily through Synopsis Activity View in Epic Aggregate responses visualized and tracked via Tableau

#### Evaluation

**Fig. 2:**
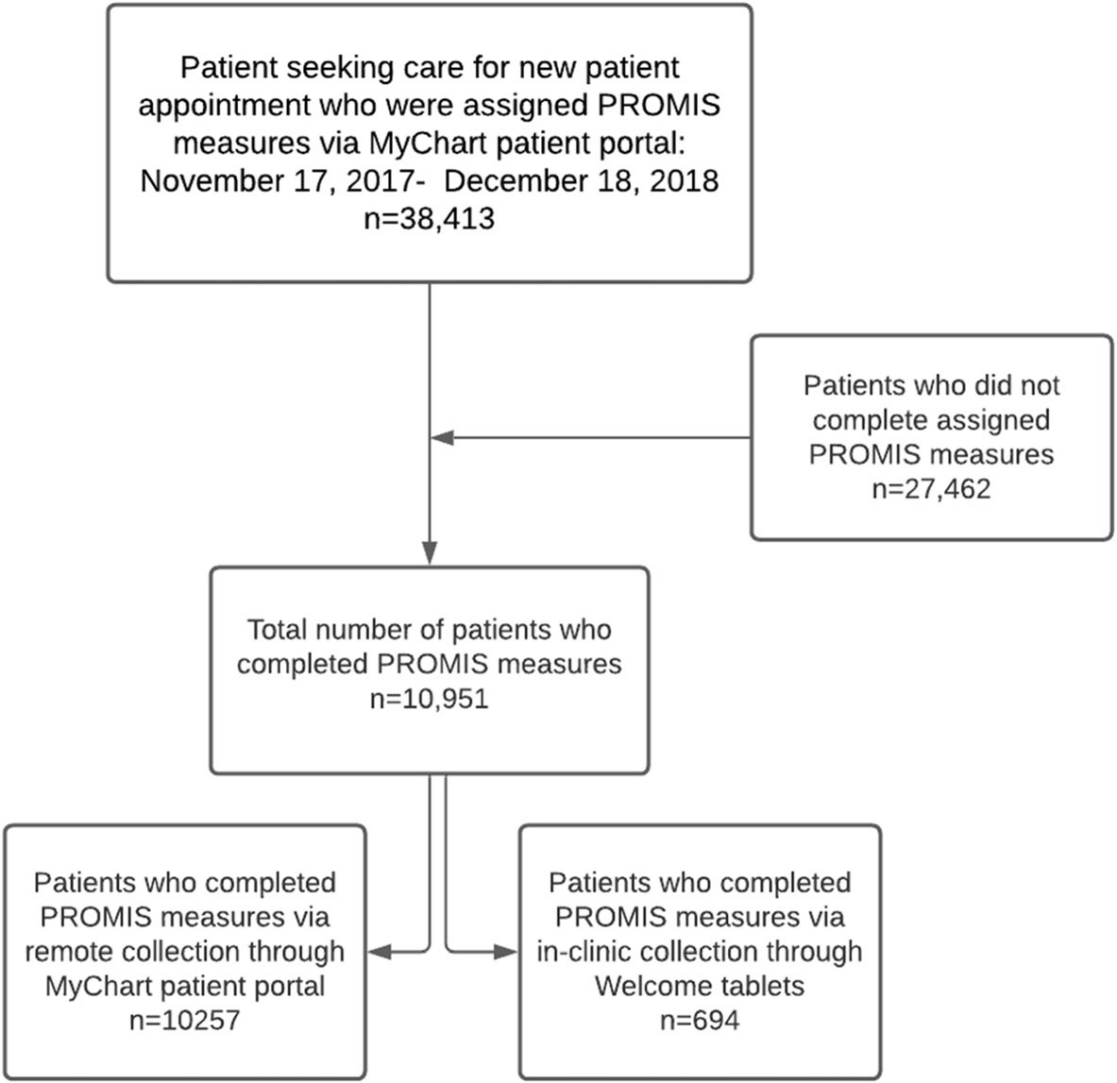
Patient eligibility flowchart

Standardized and routine PROMs administration and collection occurred from November 17, 2017, through December 18, 2018. See Fig. 2 for patient selection. The primary metric we were interested in evaluating was the response rate of patients completing PROMs. Our selected approach was a fully integrated approach that leveraged the existing capabilities of the EHR and clinical workflows. Subsequently with this approach, we were reliant on the capabilities of the local EHR, clinical sites, and personnel to collect PROMs and track in-clinic PROMs collection. The response rate to the initial PROMs assessment across the entire department was relatively low, with a 29% (*n* = 10,951) response rate for all patients and a 42% (*n* = 9162) response rate for MyChart active-only patients. However, the volume of total patients responses in the time period was substantial (*n* = 10,951). When comparing ambulatory and hospital-based clinics, the response rates were very similar. In the ambulatory clinics, the response rate for all patients was 29% (*n* = 8459), and in MyChart active-only patients, the response rate was 42% (*n* = 7072). In the hospital-based clinics, the response rate for all patients was 26% (*n* = 2492), and 41% (*n* = 2090) for MyChart active-only patients. These data are summarized in Table 4**.**

**Table 4:** Response rates for PROMs assessment

	All Patients (MyChart Active + Nonactive)	MyChart Active Patients Only
Mean Response Rate: All Clinics (*n* = 18)	29% (*n* = 10,951)	42% (*n* = 9162)
Ambulatory Clinics (*n* = 14)	29% (*n* = 8459)	42% (*n* = 7072)
Hospital-Based Clinics (*n* = 4)	26% (*n* = 2492)	41% (*n* = 2090)
Mean % for Top 5 Providers	47.3% (*n* = 526)	54.0% (*n* = 346)
Mean % for Bottom 5 Providers	14.8% (*n* = 254)	22.8% (*n* = 234)

Lastly, we sought to determine which providers had the highest initial assessment response rates as well as which ones had the lowest response rates, and assess whether there were common characteristics that were different between these groups. The mean response rate for the top 5 providers was 47.3% (*n* = 679/1426) for all patients and 54% (*n* = 652/1218) for only MyChart active patients. The mean response rate for the bottom 5 providers was 14.8% (*n* = 254/1713) for all patients and 22.8% (*n* = 252/1058) for only MyChart active patients. The provider characteristics between the top and bottom 5 patient response rates (both MyChart active patients only and all patients) differed. Regarding provider type, the top 5 were represented by primarily orthopedic surgeons, whereas the bottom 5 were mixed between orthopedic surgeons and physician assistants. Provider area of specialty differed between the top and bottom providers in that the ‘total joint’ specialty area was represented in the top 5 but not in the bottom 5. These data are summarized in Table 5**.**

**Table 5:** Characteristics of the providers with the 5 highest and lowest initial assessment response rates^a^

Provider Type	Provider Specialty	MyChart Active and Nonactive Patients Response Rate, % (n/N)	MyChart Active Patients Only Response Rate, % (n/N)
**Top 5 Providers**			
Orthopedic Surgeon	Hand	52% (178/345)	56% (167/298)
Orthopedic Surgeon	Total joint	46% (94/203)	52% 93/178
Orthopedic Surgeon	Total joint	46% (48/105)	55% (48/88)
Orthopedic Surgeon	Sports	47% (153/327)	53% (141/268)
Orthopedic Surgeon	Sports	46% (206/446)	53% (203/386)
**Bottom 5 Providers**			
Physician Assistant	Trauma	13% (20/150	23% (20/88)
Orthopedic Surgeon	Sports	15% (153/1009)	23% (152/647)
Physician Assistant	Hand	16% (12/73)	24% (12/50)
Physician Assistant	Spine	14% (45/317)	26% (45/170)
Orthopedic Surgeon	Trauma	15% (24/164)	22% (23/103)

^a^Among those with > 10 PROMs responses

## Discussion

We herein described the process of developing and implementing a fully EHR-integrated, population-based approach to PROMs collection using the thematic framework described by Gensheimer et al. [12], informed by the *Users’ Guide to Integrating Patient-Reported Outcomes in Electronic Health Records* [11]. We explored the thematic phases and addressed the 11 key questions outlined by Gensheimer et al. to determine the implementation approach for PROMs collection in our department. Lastly, we performed an evaluation of our implementation approximately 13 months after beginning the initiative to determine the scope, scale, and uptake of our PROMs collection effort. We found that in following the framework, we were able to develop an implementation strategy that met the goal of standardized PROM collection in our department as part of the strategic plan while also leveraging the existing resources of our local EHR to maximize clinical utility of PROMs and routine collection. With our initial implementation approach, we achieved a large scale of routine collection across 18 clinical sites. The scope of our initiative was broad, covering 7 clinical specialties and capturing outcomes related to visits to orthopedic surgeons, non-operative orthopedic providers, and advanced practice providers (nurse practitioners and physician assistants). Our response rate to the initial questionnaires varied across the department, with the highest single provider response rate reaching an average of 52% and the lowest provider 13%. The response rates for the department were fairly consistent between hospital-based and ambulatory clinics, but differences were seen consistently for MyChart active only versus all patients, where response rates were higher for the MyChart active patients. This is to be expected, as our PROMs collection initiative primarily relied on the use of the patient portal (MyChart). Some clinics had the Welcome tablets available, but they were not consistently used and only accounted for an 6% overall percentage of initial PROMs assessments collected.

Given substantial changes in the data “ecosystems” that support the capture and use of patient-reported outcomes in health care delivery [18], it is important to identify and understand barriers to and facilitators of the successful implementation of PROMS collection [19]. The decision-making process and challenges we faced with our initiative are similar to other studies reporting the implementation of PROMs collection. With our implementation approach we were able to successfully administer, collect, and report PROMs within the EHR and through data visualization applications as other studies in orthopedic settings have demonstrated [20–22]. Moreover, we decided to collect PROMIS measures as our standard measure, as opposed to region-specific or legacy measures associated with each clinical division, which allowed us to scale the collection of PROMs across our department while minimizing the complexity of EHR programming for administering and reporting our PROMs. Our approach and findings adds to the body of literature supporting use of PROMIS measures as a routinely collected set of measures in Orthopaedics [23, 24]. By integrating PROMs collection into the EHR and clinical care, we made PROMs data available and actionable for providers for clinical decision-making, to assess the patient’s experience of his or her symptoms or illness, in addition to objective findings [25]. The challenges we faced were consistent with the themes reported in other studies such as logistical and workflow issues, technological barriers, and patient engagement and activation [26, 27]. Our most notable challenge was increasing our overall response rate for PROMs collection; our rate for all patients (29%) was consistent with the statistic for general surveys (33%) [28], and our rate was slightly higher for patients who were MyChart active (42%). The variation we reported in our study is consistent with other PROMs collection initiatives reported in orthopedics (34–77% over a 1-year period [20]. However, the general response rate for PROMs collection initiatives in orthopedics appears to hover around 70–80%, with studies reporting 74.9% [29] and 72.6% [30], and the UK National Joint Registry reporting 64%, the Swedish Hip Arthroplasty Registry 79%, and the New Zealand Joint Registry 70% [31]. A notable difference between our initiative and the above-mentioned studies is that we employed a population-health based approach for PROMs collection solely within the EHR (fully integrated approach), where as these studies collected PROMs via outside collection platforms that rely on email correspondence rather than patient portals for administration. Our response rate (42%) is similar to that of another study that administered PROMIS measures through MyChart (37%) [32], confirming that the mode of administration may affect the response rates. A noted difference in our study compared to similar studies is that we scaled our PROMs collection across a large department (18 clinics) and many specialties (7 specialties). Many of the previous studies of PROMS collection were primarily conducted in one orthopedic patient population or clinical specialty, involved data capture outside the EHR, or had a targeted recruitment strategy with dedicated personnel responsible for achieving response rates of 80% [33]. We faced similar challenges as other studies employing remote PROM collection that reported low response rates (24–36%) [34–37]. This indicates that perhaps response rates for PROM collection embedded in clinical care are expected to be lower unless resources are provided to increase the rate; however, these approaches have the potential to achieve a higher volume of responses that result in more actionable data at the point of care [38, 39]. The two major barriers that we faced in improving the response rates was the reliance on the EHR to administer and collect PROMs without consistent in-clinic collection strategies and the lack of real-time monitoring of response rates to provide feedback to clinical sites on performance.

The implementation of our PROMs collection has some strengths and limitations. First, the major strengths of our study were the scale and scope of our initiative. By leveraging the existing capabilities of the EHR, we were able to collect a large volume of patient responses over a 13-month period (*n* = 10,951) with minimal effort by clinicians or staff, and patients were able to complete the questionnaires at their leisure. Moreover, we collected a diverse set of PROMIS measures, allowing us to make future determinations about managing our patient population and inform subsequent treatment decisions using historical patient data. The limitations of our initiative were primarily related to clinical workflow issues, tablet availability, and provider engagement with PROMs collection. Some clinical sites reported difficulties with determining which patients still needed to complete their PROMs. Moreover, each clinical site differed in the clinical workflow; therefore, one approach for PROMs collection was not feasible. During our implementation period, welcome tablets were not available at most sites. Additionally, the Epic Welcome module was a fairly new feature, and there were frequent technological issues with the Welcome tablets, resulting in inconsistent use. Lastly, continued provider engagement was challenging. During the selection process, we selected a generic PROM based on clinician feedback. However, during the implementation period, many clinicians reported still wanting to collect legacy measures or difficulty interpreting PROMIS measures.

## Conclusions

We demonstrated that PROMs can be successfully collected at scale through the EHR across a large department with seven clinical orthopaedic specialties. However, we did encounter similar logistical, technical, and engagement issues as have other studies reporting PROMs collection relying primarily on remote collection [26, 27]. The findings of our study add value to the ecosystem of PROMs collection. We are the first study we are aware of to successfully administer standardized PROMIS measures across an entire department with a heterogeneous patient population through primarily remote EHR collection. This study has yielded valuable information regarding the potential trade-off between implementing a scalable, population health based approach and achieving adequate response rates. Therefore, future steps for PROMs collection should focus on improving the robustness of PROMs response rate by updating utilities within the EHR that improve communication with patients and demonstrate how PROMs support shared decision-making with providers.

## Data Availability

The datasets generated and/or analysed in this study are not publicly available and restrictions apply to the availability of these data. Data may be requested from the authors upon reasonable request and only after the conclusion of all analyses pertaining to this data.

## Abbreviations

PROMs: patient reported outcome measures
EHR: electronic health record
IT: information technology
PROMIS: patient reported outcome measurement information system
COORDS: comprehensive outcomes in orthopaedics and rehabilitation data system
